# Design of a Novel Conditional Noise Predictor for Image Super-Resolution Reconstruction Based on DDPM

**DOI:** 10.3390/jimaging11050138

**Published:** 2025-04-29

**Authors:** Jiyan Zhang, Hua Sun, Haiyang Fan, Yujie Xiong, Jiaqi Zhang

**Affiliations:** School of Software, Xinjiang University, Urumqi 830091, China; 18099202351@163.com (J.Z.);

**Keywords:** deep learning, image super-resolution reconstruction, DDPM, conditional noise predictor

## Abstract

Image super-resolution (SR) reconstruction is a critical task aimed at enhancing low-quality images to obtain high-quality counterparts. Existing denoising diffusion models have demonstrated commendable performance in handling image SR reconstruction tasks; however, they often require thousands—or even more—diffusion sampling steps, significantly prolonging the training duration for the denoising diffusion model. Conversely, reducing the number of diffusion steps may lead to the loss of intricate texture features in the generated images, resulting in overly smooth outputs despite improving the training efficiency. To address these challenges, we introduce a novel diffusion model named RapidDiff. RapidDiff uses a state-of-the-art conditional noise predictor (CNP) to predict the noise distribution at a level that closely resembles the real noise properties, thereby reducing the problem of high-variance noise produced by U-Net decoders during the noise prediction stage. Additionally, RapidDiff enhances the efficiency of image SR reconstruction by focusing on the residuals between high-resolution (HR) and low-resolution (LR) images. Experimental analyses confirm that our proposed RapidDiff model achieves performance that is either superior or comparable to that of the most advanced models that are currently available, as demonstrated on both the ImageNet dataset and the Alsat-2b dataset.

## 1. Introduction

Computer vision stands as a pivotal branch of artificial intelligence, playing a crucial role in its implementation. Within the domain of image super-resolution (SR) research and application, image SR emerges as a significant area of focus, with the objective of reconstructing high-resolution (HR) images from one or multiple low-resolution (LR) inputs. This process aims to enhance both the quality and detailed features of the resulting images. Image SR possesses extensive application value across various fields, including surveillance video analysis, medical image processing, satellite image assessment, facial recognition, and the restoration of old photographs.

Early methods for image super-resolution (SR) reconstruction primarily relied on image self-similarity [1] and sparse representation techniques [2]. The image self-similarity approach exploits the inherent positional similarities present within the image at varying scales to effectively reconstruct its details and structures. On the other hand, sparse representation capitalizes on the sparse characteristics of an image within specific transformation domains to extract prominent features, thereby aiding the image reconstruction process. Nevertheless, these methods demonstrate a substantial dependence on the self-similarity or sparsity of images, limiting their applicability in numerous scenarios.

In contrast, deep learning has revolutionized the field of computer vision with its powerful feature representation capabilities, gradually supplanting traditional interpolation and filtering-based methods. Consequently, deep learning-based image super-resolution techniques have become mainstream. Notably, Dong et al. [3] were among the pioneers in applying convolutional neural networks (CNNs) to image SR reconstruction, leading to a continuous stream of innovations and the emergence of various novel network architectures. Examples include enhanced algorithms for SR convolutional neural networks [4] and reconstruction methods utilizing residual networks, as proposed by Kim et al. [5], as well as approaches based on dense connections, introduced by Ahn et al. [6]. These early methodologies primarily aimed to optimize the peak signal-to-noise ratio (PSNR), often resulting in reconstructed images that appeared overly smooth, thereby failing to capture the intricate texture details. This limitation prompted the advent of generative models in this field. Currently, the mainstream generative models employed to address SR tasks are based on generative adversarial networks (GANs) and diffusion models, both of which place a greater emphasis on the visual quality and structural similarity of the reconstructed images.

The image super-resolution technique informed by generative adversarial networks (GAN) operates through a dynamic zero-sum game involving a generator and a discriminator. The generator is tasked with learning the authentic distribution of the images to produce realistic outputs, while the discriminator assesses the veracity of the images, discerning whether they are generated or genuine. This iterative process continues until the discriminator is unable to distinguish between real and generated images, resulting in a Nash equilibrium state for the model. Notably, Ledig et al. [7] pioneered the application of GANs to image SR tasks through their development of the SR generative adversarial network (SRGAN), which demonstrated superior visual effects when compared to traditional PSNR-based image SR approaches. However, this method was criticized for producing artifacts within the reconstructed images. Subsequent research sought to improve the network and framework of the GAN based on this foundation to achieve better results. Wang et al. [8] proposed using prior knowledge of images and relative discriminators to improve the performance of SRGAN in reconstructing image features, enriching the detailed features of image generation. However, this method overly relies on prior knowledge of the image, and, when there is insufficient or unsuitable prior knowledge, the details and features of the reconstructed image will be significantly reduced. Indradi et al. [9] proposed introducing residual modules to improve the quality of images generated by GANs, but this method shows a decrease in learned perceptual image patch similarity (LPIPS). Although the aforementioned GAN-driven image SR methods can reconstruct images with more detailed features, they still face some problems, such as unstable training and a lack of diversity in the generated images.

The denoising diffusion probabilistic model (DDPM) [10], a generative model, recently presented good performance in the field of image SR tasks. The image SR method based on diffusion models is a method in which the diffusion model first adds Gaussian noise to the image during the forward diffusion process. It then learns the backward diffusion process through the denoising model, allowing for the acquisition of the original image’s distribution. The design of the loss function in the DDPM is simple and provides sufficient theoretical support for stable training.

In the traditional DDPM, image restoration is achieved through a gradual denoising process, which helps to avoid common issues such as over-sharpening or artifact generation that are typically seen in super-resolution models. The images generated by the DDPM tend to exhibit more natural textures and finer details compared to generative adversarial networks (GANs). The DDPM’s process also ensures higher sample diversity, making it especially suitable for tasks such as image restoration, super-resolution, and noise removal, producing smoother and more stable images with fewer artifacts.

However, while the DDPM offers significant advantages over GANs, it does have notable limitations. One of the main drawbacks lies in its generation speed, which is often compromised due to the model’s need to perform multiple sampling steps. Each step involves computing the model’s output and making subtle adjustments to the generated image, which increases the computational demands and slows down the overall generation process. This is particularly problematic in high-resolution image generation, where the need for fine-tuning at each step can result in substantial memory usage and an increase in the hardware load. As the number of diffusion steps increases, the computational resources required increase and the training efficiency decreases; this significantly degrades the overall performance, especially when dealing with high-resolution images.

Another issue with the DDPM is the misalignment of high-frequency details in the generated images. The conditional noise predictor (CNP) in the DDPM, which typically uses a U-Net structure, tends to focus on high-frequency components linked to local features of the image, while neglecting the global semantic information that is crucial for accurate image generation. In real-world scenarios, however, images are composed of both high-frequency and low-frequency signals. This over-reliance on local information leads to inconsistent high-frequency details, causing noise with significant variance in the noise prediction process and ultimately resulting in unwanted textures in the reconstructed images.

Addressing these challenges—particularly the alignment of high-frequency details with global image semantics and improving the generation speed—remains an important issue in super-resolution tasks. The main contributions of this article are as follows.

(1) This study aims to bridge the semantic gap between the encoder and decoder in DDPMs, which is a fundamental challenge when using the U-Net-based CNP. The key goal of this work is to enhance the noise prediction ability of the CNP by integrating a dual-decoder architecture. By combining a U-Net decoder with a Transformer decoder, the proposed model ensures that both local (high-frequency) and global (low-frequency) information is effectively utilized. This innovation allows the model to reduce the number of diffusion steps needed during image generation, thus improving the inference speed while preserving the image quality.

(2) The novel contribution of this study is the development of the dual-decoder CNP, which improves noise prediction by incorporating both U-Net and Transformer decoders. This dual-decoder structure compensates for the semantic gap between the encoder and decoder, ensuring that the generated super-resolution images retain both clear details and texture fidelity. This approach not only enhances the quality of the generated images but also improves the model’s adaptability and stability across different input conditions, making it more efficient and effective for a wider range of remote sensing and image restoration tasks.

## 2. Related Work

### 2.1. Recent Advances in Image Super-Resolution

The goal of image SR is to restore the HR images from the degraded LR images [11]. Early SR methods assumed a degradation process, such as downsampling or adding a fuzzy kernel [12]. Although this method has good performance on synthetic data with the same degradation, its limited generalization makes it difficult to recover good detail features in practical real-world scenarios [13].

As noted by Chen et al. [14], the focus of recent research has shifted from degradation synthesis settings to SR with unknown degradation. Because of the lack of paired HR data during the training process, some tools adopt unsupervised methods to implicitly learn degraded models from LR, such as CycleGan [15]. In addition to unsupervised learning, other methods aim to display synthetic real-world image pairs and learn degradation models from them. Specifically, BSRGan [16] and Real-ESRGan use degradation pipelines to achieve real-world data pairing. Based on the use of degradation pipelines to provide data pairing, recent works based on diffusion models [17] further show competitive performance in real-world image SR. One common method is to retrain the diffusion model from scratch by concatenating the LR image with the noise in each step. Another common approach is to apply the reverse process by adding constraints and using an unconditional pre-trained diffusion model as a prior. To produce a realistic HR image, both approaches frequently require hundreds or thousands of sample steps.

### 2.2. Diffusion Model

**Figure 1:**
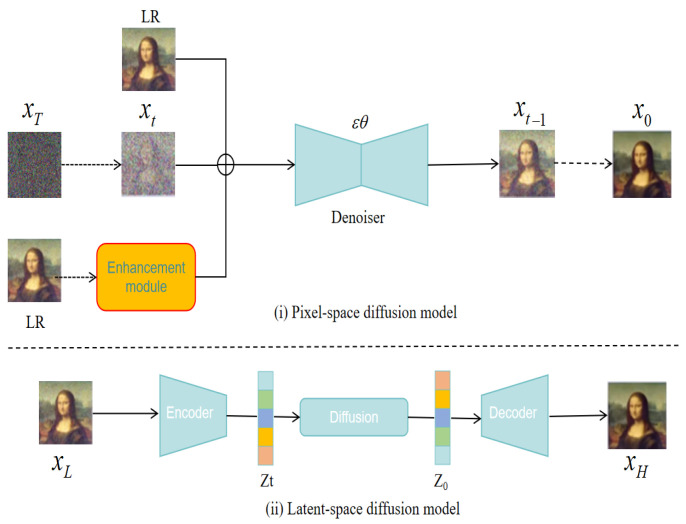
Network architectures of the pixel-level spatial diffusion model and latent spatial diffusion model.

As a type of generative model, diffusion models have attracted global attention due to their powerful feature expression abilities. Examples include the currently popular pixel-level spatial diffusion model and latent spatial diffusion model, as shown in Figure 1. Recent years have seen the proposal of diffusion models for a variety of high-level image manipulation tasks, such as conditional or unconditional generation, text-to-image translation [18], image super-resolution [19], and others. First, Song et al. [20] presented a score-based model that uses gradients of the data distribution determined with score matching to generate samples via Langevin dynamics. The equivalence of the DDPM proposed by Ho et al. [10] from the direction of the weighted variational bound is demonstrated in [20]. The DDIM [21] uses a non-Markov-chain sampling procedure to speed up the sampling of the DPM. Furthermore, by calculating the linear portion of the ODE and using a change in variables, DPM-solver [22] expedites the sampling process by simplifying the solution to an exponentially weighted integral of the neural network. Meanwhile, EDM [23] separates several diffusion model design components and creates a second-order ODE sampler to liberate the DPM design from mathematical limitations. This further enhances the diffusion model to achieve state-of-the-art performance.

Furthermore, GAN-based and flow-based models (apart from diffusion models) constitute the majority of generative approaches. GAN-based models [7,24,25] use a discriminate model to create a sample based on a data distribution. Its function is to estimate the likelihood that a sample originates from the training data instead of the generative model. Meanwhile, by converting a basic input distribution (such as Gaussian) into the target distribution via a sequence of invertible transformations, flow-based models [26] discover the underlying distribution of the data. Diffusion models are able to produce images with greater fidelity and more information than the two generative models previously described. Diffusion models provide the advantages of low mode collapse, stable training, and training with a single mean squared error (MSE) loss. Diffusion models are simpler to train and build than GAN-based models, which have instability problems during adversarial training, and flow-based models, which have network performance limitations because of the reversibility requirement.

The recently proposed DDPM-based SR model is used to generate detailed texture information in image generation tasks. For example, SRDiff uses LR encoders to replace LR images with feature maps extracted from residual dense blocks (RRDB). LDM diffuses in latent space, reducing the computational complexity of the reconstructed images while improving their quality. However, the excessive utilization of high-frequency information related to local features may result in inaccurate details in DDPM-based models, while the U-Net-based CNP may generate noise with significant variance. Our model is based on a residual diffusion network framework, which solves this problem by introducing a Transformer decoder and feature fusion block in the CNP. This model combines global contextual relationships to bridge the semantic gap between the encoder and decoder. Through its enhanced dual-decoder design, our model can generate detailed information while maintaining image accuracy.

In summary, the current image SR reconstruction methods aim to predict the pixel similarity between reconstructed images and real images. Therefore, although the predicted image has high image quality, these methods often result in issues of excessive smoothing and the inaccurate restoration of detailed features. This fails to satisfy the image quality requirements for the application of SR in downstream tasks such as medical image processing and facial recognition.

Therefore, in order to resolve these issues, we propose an effective diffusion model in this paper. This model improves the semantic gap between the decoder and encoder by using a dual-decoder structure design, maximally preserving the feature information of LR images and ultimately generating SR images with more details and texture features.

## 3. Methodology

### 3.1. RapidDiff Design

**Figure 2:**
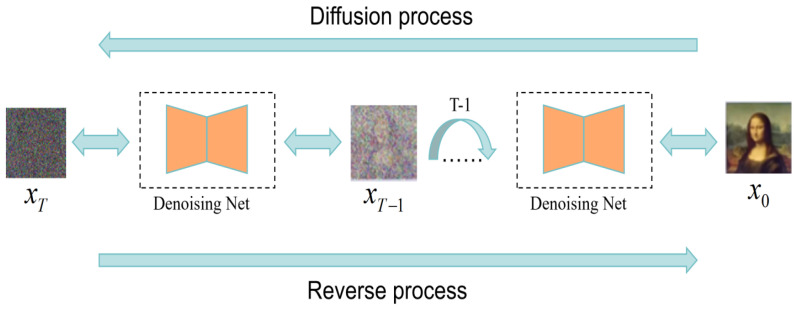
The structure of the RapidDiff model. Image SR reconstruction is performed using two Markov chains constructed by moving the residuals between HR and LR.

Based on the DDPM, we propose a RapidDiff model consisting of two Markov chains, as shown in Figure 2. One of the forward chains is used for the diffusion step, which aims to diffuse the distribution of real-world image data into a random noise data distribution. A reverse chain is used for reverse steps; the purpose of the reverse chain is to transform the distribution of random noise data into real-world image data.

(1) Diffusion process

Given the original image $x_{0}$, a sequence of data distributions {$x_{1}$, $x_{2}$, $x_{3}$,…, $x_{T}$} is generated by adding random noise through the diffusion steps *T* from $x_{0}$. $q\left(x_{t}|x_{t-1}\right)$ is the conditional probability that defines how the state evolves from $x_{t-1}$ to $x_{t}$. The diffusion process is as follows:

(1) $$q\left(x_{1},\ldots ,x_{T}|x_{0}\right)=\prod_{t=1}^{T}q\left(x_{t}|x_{t-1}\right)$$

(2) $$q\left(x_{t}|x_{t-1}\right)=N\left(x_{t};x_{t-1}+\alpha_{t}\gamma_{0};\kappa^{2}\alpha_{t}I\right),t=1,\ldots ,T$$

Equation (2) represents the transition probability in a diffusion process, where each state $x_{t}$ is a Gaussian distribution centered around a noisy version of the previous state $x_{t-1}$. Here, $\gamma_{0}=x_{0}-y_{0}$ represents the residual between HR and LR; $x_{0}$ and $y_{0}$, respectively, represent an HR image and LR image. $\alpha_{t}=\eta_{t}-\eta_{t-1}$ in Equation (2), where $\eta_{t}$ represents a shifting sequence, which monotonically increases with time step *T*. When t=0, $\eta_{t=0}$ approximates 0, and, when t=T, $\eta_{t=T}$ approximates 1. κ, N, and I are, respectively, a hyperparameter that controls the variance of noise, a Gaussian distribution, and an identity matrix. In general, every $x_{t}$ can be inferred and calculated by $x_{t-1}$, indicating that $q\left(x_{t}|x_{0}\right)$ can be calculated as follows:

(3) $$q\left(x_{t}|x_{0}\right)=N\left(x_{t};x_{0}+\eta_{t}\gamma_{0};\kappa^{2}\eta_{t}I\right),t=1,2,\ldots ,T$$

Equation (3) describes the conditional probability distribution of a state $x_{t}$ at time step *t* given the initial state $x_{0}$ in a diffusion process. At each time step, the state $x_{t}$ is generated by adding noise to the initial state $x_{0}$, modeled by a Gaussian distribution. Therefore, according to mathematical induction, each $x_{t}$ can be calculated from $x_{0}$ using the following expression:

(4) $$x_{t}=x_{0}+\eta_{t}\gamma_{0}+\kappa \sqrt{\eta_{t}}\xi_{t}$$

Equation (4) describes the evolution of the state $x_{t}$ at time step *t* in a diffusion process, where the state evolves from an initial condition $x_{0}$ by adding noise. There are two components: $\eta_{t}\gamma_{0}$ represents the transition of the residual between HR and LR at time *t*; $\kappa \sqrt{\eta_{t}}\xi_{t}$ represents the random noise at time *t*, where $\xi_{t}$ ~N(0,I).

(2) Reverse process

The reverse process aims to convert data from $p_{\theta }\left(x_{T}\right)$ to $p_{\theta }\left(x_{0}\right)$, parameterized by θ. The prior distribution is chosen by $p\left(x_{t}\right)=N\left(x_{t};0,I\right)$ since $q(x_{t})$ approximately follows $N\left(x_{t};0,I\right)$ in the diffusion process. Therefore, the process of gradually transitioning from $x_{t}$ to $x_{0}$ can be represented by the following equation:

(5) $$p_{\theta }\left(x_{0},\ldots ,x_{T-1}|x_{T}\right)=\prod_{t=1}^{T}p_{\theta }\left(x_{t-1}|x_{t}\right)$$

Equation (5) represents how the joint probability of the sequence {$x_{0}$, $x_{1}$, $x_{2}$,…, $x_{T-1}$}, given $x_{T}$, can be factorized into a product of conditional probabilities. $p_{\theta }\left(x_{0},\ldots ,x_{T-1}|x_{T}\right)$ is the conditional probability of the sequence {$x_{0}$, $x_{1}$, $x_{2}$,…, $x_{T-1}$} given the final state $x_{T}$. In other words, it represents the probability of the sequence $x_{0}$ through $x_{T-1}$, assuming that we already know $x_{T}$. $\prod_{t=1}^{T}p_{\theta }\left(x_{t-1}|x_{t}\right)$ is a product of the conditional probabilities. Each term $p_{\theta }\left(x_{t-1}|x_{t}\right)$ represents the probability of $x_{t-1}$ given $x_{t}$. This conditional probability describes how we can infer $x_{t-1}$ from $x_{t}$ at each time step. The process of reversal from $x_{t}$ to $x_{t-1}$ is as follows:

(6) $$p_{\theta }\left(x_{t-1}|x_{t}\right)=N\left(x_{t-1};\mu_{\theta }\left(x_{t},t\right),\sigma_{\theta }{\left(x_{t},t\right)}^{2}I\right)$$

Equation (6) is a conditional probability distribution in the context of diffusion models. $p_{\theta }\left(x_{t-1}|x_{t}\right)$ represents the conditional probability of $x_{t-1}$ given $x_{t}$. Here, θ, $\mu_{\theta }$, and $\sigma_{\theta }$ are, respectively, the model parameters, mean, and variance, which follow a Gaussian distribution. $\mu_{\theta }\left(x_{t},t\right)$ is a function that predicts the most likely value of $x_{t-1}$ based on current state $x_{t}$ and time step *t*, while the variance $\sigma_{\theta }{\left(x_{t},t\right)}^{2}$ quantifies the uncertainty in this prediction. The optimization of the θ parameter is achieved by minimizing the negative evidence lower bound, namely

(7) $$\min_{\theta }\sum_{t}D_{\mathrm{KL}}\left[q\left(x_{t-1}|x_{t},x_{0},y_{0}\right)∥p_{\theta }\left(x_{t-1}|x_{t},y_{0}\right)\right]$$

Equation (7) represents the objective to minimize the difference between the true posterior distribution $q\left(x_{t-1}|x_{t},x_{0},y_{0}\right)$ and the predicted distribution $p_{\theta }\left(x_{t-1}|x_{t},y_{0}\right)$ for each time step *t*. By minimizing the KL divergence, the model learns to effectively denoise, generate data, and better predict the previous state $x_{t-1}$ from the noisy state $x_{t}$. For the sake of simplicity in calculation, after ignoring terms unrelated to θ, the loss function ζ can be simplified as shown in the following formula:

(8) $$\min_{\theta }\zeta_{t-1}=E_{x0,\varepsilon ,t}\left[{∥\varepsilon -\varepsilon_{\theta }\left(x_{t},t\right)∥}^{2}\right]$$

Equation (8) aims to minimize the loss function $\zeta_{t-1}$, which is based on the mean squared error (MSE) between the true noise ε and the model’s predicted noise $\varepsilon_{\theta }\left(x_{t},t\right)$. Minimizing this loss $\zeta_{t-1}$ during training helps the model to improve its ability to denoise data at each time step. Here, $\varepsilon_{\theta }$ is the noise generator that we propose. Through the loss function ζ, we calculate the residual between the random noise and our predicted noise. Using the Adam optimizer to optimize and reduce the residual between the random noise and our predicted noise, we can gradually recover from $x_{t}$ to $x_{0}$. Finally, if we assume ξ ~N(0,I), we can represent the process of restoring from $x_{t}$ to $x_{t-1}$ as follows:

(9) $$x_{t-1}=\mu_{\theta }\left(x_{t},t\right)+\sigma_{\theta }{\left(x_{t},t\right)}^{2}\xi_{t}$$

Equation (9) indicates that the model generates a cleaner sample $x_{t-1}$ from a noisy sample $x_{t}$ at time step *t*. The model uses the predicted mean $\mu_{\theta }\left(x_{t},t\right)$ and variance $\sigma_{\theta }{\left(x_{t},t\right)}^{2}$ to denoise, while the random noise ξ is added to maintain the stochastic nature of the generation process. This step is essential in recovering clean data from noise in the reverse diffusion process.

### 3.2. Conditional Noise Predictor

**Figure 3:**
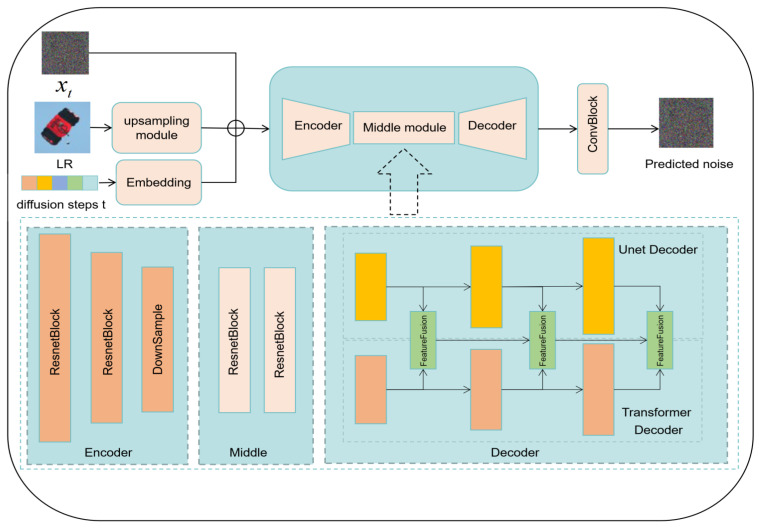
The structure of the conditional noise predictor for the RapidDiff model.

The aim of the CNP is to learn the noise in the process of HR spreading to LR during the training process and then predict the noise to restore LR to SR during the inference stage. Sui et al. [27] showed that the CNP can effectively predict corresponding noise through random noise, thereby improving the ability to recover detailed features in image SR reconstruction tasks. The specific structural design of the CNP is shown in Figure 3.

The CNP is mainly composed of three parts: the encoder module, the middle module, and the decoder module. At the beginning, $x_{t}$, LR is processed by the upsampling module, embedding high-dimensional sparse diffusion time steps into low dimensional dense data through position encoding. Then, the data are sent to the encoder module for coarse feature extraction. Next, the features extracted from the encoder module will be passed through the middle module, consisting of two ResNet blocks. Lastly, the decoder module receives the output from the intermediate module. The decoder module is composed of a U-Net decoder and a Transformer decoder, which are connected to each other through a feature fusion block. The feature fusion module weights the feature map channels based on global information, which can enhance useful features and suppress unimportant features, thus achieving the accurate extraction of feature information.

#### 3.2.1. Encoder Module

The encoder first creates a multi-layer perceptron (MLP), consisting of a sine position encoding module, two fully connected layers, and an activation function, ReLu. The diffusion time step *t* is first processed through sine position encoding and the MLP to obtain t_emb. Next, the features obtained by processing the LR image through an upsampling module are deconvolved and extracted to form cond. We conduct advanced image feature extraction on t_emb, $x_{t}$, and cond by applying two residual network modules (ResNet blocks) and one downsampling module (downsample block) and then store the extracted results for use as input in the decoder.

#### 3.2.2. Decoder Module

In our design, the U-Net decoder and Transformer decoder each have different advantages. The U-Net decoder performs well in image generation and restoration tasks, especially when capturing local details and low-level features. It can effectively preserve high-resolution feature information through skip connections, thereby helping to restore the details of the image. On the other hand, Transformer decoders are adept in capturing global dependencies and can handle long-distance information transmission, especially when dealing with complex structures and contexts. The Transformer can model in a wider range of contexts through its self-attention mechanism. The purpose of combining these two is to fully utilize their respective advantages: U-Net provides the ability to recover local details, while the Transformer enhances the modeling ability of global information. Through this fusion, we expect to obtain more accurate outputs, especially in tasks that require the simultaneous consideration of local features and global contexts. We believe that integrating the outputs of the U-Net decoder and Transformer decoder has significant advantages. By combining the outputs of these two, we can obtain a decoder that strikes a balance between spatial accuracy and global context modeling. This fusion helps to improve the performance of the model, especially in complex tasks such as generating high-quality images with consistent details and structures. While merging two decoder modules may render the architecture more complex, this complexity is carefully considered, and its effectiveness has been demonstrated in ablation experiments. We noted the performance improvement brought by this fusion during the design process, especially in capturing details and global structures in the image. If only a single decoder module is used (e.g., only the U-Net or Transformer decoder), the advantages of both may not be fully utilized in some tasks, resulting in performance degradation. Therefore, although the architecture may appear more complex, we believe that this design is necessary; from the results, the performance improvement brought about by the complexity is worthwhile.

The decoder module is composed of two parts: the U-Net decoder and the Transformer decoder. The U-Net decoder consists of two residual network modules (ResNet block) and one upsampling module (upsample block). t_emb provides guidance for the calculation of high-frequency feature semantic information throughout the entire U-Net decoder. The Transformer decoder uses a module, DitBlock, that combines a self-attention mechanism and MLP and employs adaptive normalization to support the adjustment of input $x_{t}$ by t_emb. Next, a weighted fusion module, WFBlock, is used to upsample the input features and adjust the number of feature channels. After the weighted fusion of the two feature maps using weight parameters, batch normalization and ReLu activation processing are performed.

#### 3.2.3. Feature Fusion Block

**Figure 4:**
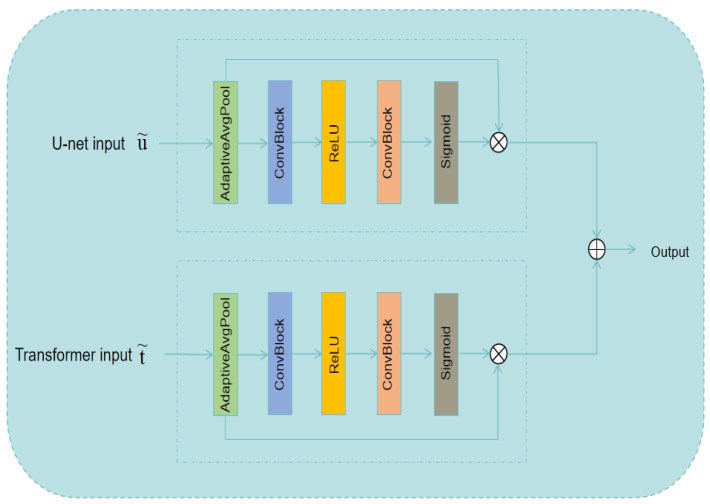
The architecture of the feature fusion block.

The feature fusion block is a squeezable module based on a CNN that increases the expressive power of feature maps by learning channel weights. As shown in Figure 4, the output of each layer of the Transformer decoder and U-Net decoder is passed through an adaptive average pooling layer to pool each channel of the input feature map into a single scalar. Next, the dimensionality is reduced through one kernel convolution; it is then activated by the ReLu activation function and restored to its original dimensions through one kernel convolution before undergoing sigmoid activation processing. Finally, the processed outputs of the dual decoders are added and fused for feature fusion as the final output of the feature fusion block. The calculation method of the feature fusion block can be expressed as

(10) $$u_{i}={\tilde{u}}_{i-1}+FeatureFusionBlock\left({\tilde{u}}_{i-1},{\tilde{t}}_{i-1}\right)$$

(11) $$t_{i}={\tilde{t}}_{i-1}+FeatureFusionBlock\left({\tilde{u}}_{i-1},{\tilde{t}}_{i-1}\right)$$

In this context, let ${\tilde{t}}_{i}$ and ${\tilde{u}}_{i}$ denote the output results of the *i*-th layer of the U-Net decoder and the Transformer decoder, respectively. Conversely, $t_{i}$ and $u_{i}$ represent the corresponding inputs of the *i*-th layer for the U-Net decoder and the Transformer decoder, respectively.

#### 3.2.4. Training and Inference

**Figure 5:**
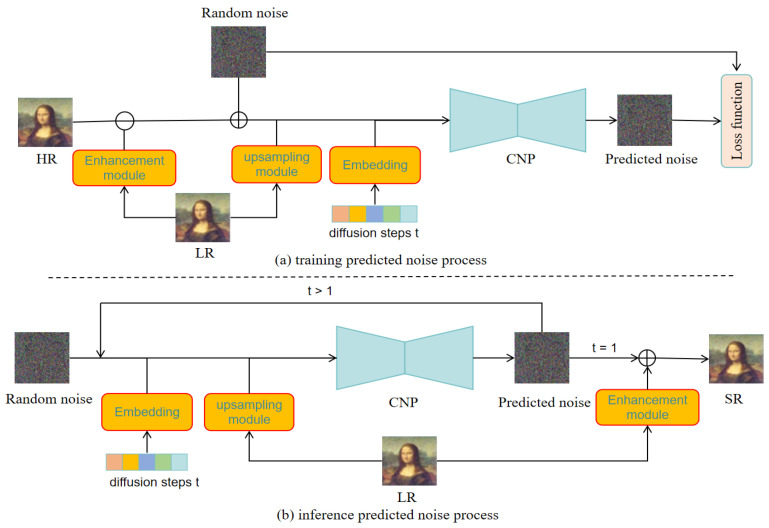
The process of inference and training with predicted noise.

As illustrated in Figure 5a, during the training phase, focused on noise prediction, we compute the residual between the high-resolution (HR) image and the low-resolution (LR) image that has undergone processing through the enhancement module. Subsequently, we generate a noisy dataset by introducing random noise to this residual. Then, the noise data, diffusion steps *t*, and LR features obtained through the upsampling module are sent together to the CNP for noise prediction. The diffusion steps t and random noise are, respectively, noise that follows a standard Gaussian distribution and a sequentially increasing set of integers {1,…,T}. Finally, we construct a loss function based on Equation (8) to calculate the minimum negative evidence lower bound for the optimization of the CNP.

As shown in Figure 5b, during the inference noise prediction process, we first generate random noise that conforms to a Gaussian distribution, representing the current diffusion time step *t*. At each diffusion time step *t*, the features extracted by upsampling from LR are fed into the CNP along with the random noise at the current diffusion time step for noise prediction. When t>1, the loop will continue to combine the predicted noise obtained with the features extracted by LR through upsampling and send them to the CNP for noise prediction. When t=1, the reconstruction step from $x_{t}$ to $x_{0}$ will be completed using Equation (9) and the predicted noise obtained. Finally, $x_{0}$ and the enhanced images of LR through the enhancement module are added together as the output of the final SR task.

## 4. Experiments

This section introduces the performance parameters and experimental results of the RapidDiff model and demonstrates its effectiveness on a synthetic dataset (ImageNet [28]) and a remote sensing dataset (Alsat-2b [29]).

### 4.1. Dataset Introduction

**Figure 6:**
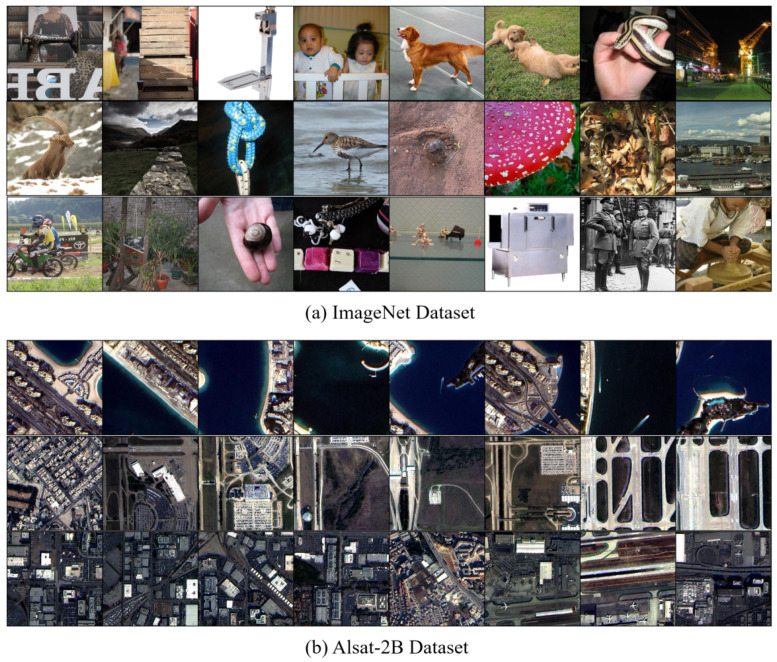
A partial example of the ImageNet dataset and the Alsat-2B dataset.

The specific parameters of the ImageNet dataset and Alsat-2B dataset are shown in Table 1. The content shown in Figure 6 is a partial example of the ImageNet dataset and the Alsat-2B dataset.

ImageNet is a large visualization database used for research on computer vision. It has been collected and produced by the team led by Feifei Li since 2007, using various methods, such as web scraping, manual annotation, and Amazon crowdsourcing. It was published as a paper in CVPR-2009. The ImageNet dataset contains 1000 categories. The HR images in the training and testing sets of ImageNet are randomly cropped to a size of 256 × 256, while the LR images are degraded by a degradation model on the HR images.

Alsat-2B is a collection of remote sensing images that encompasses both low and high spatial resolutions, specifically designed for single image super-resolution (SR) tasks. The high-resolution images within this dataset are generated using pan sharpening techniques. This dataset is derived from images taken by the Alsat-2B Earth observation satellite. The Alsat-2B remote sensing dataset covers 13 different cities and encompasses various landforms, such as farmland, towns, islands, mountainous areas, and airports.

### 4.2. Experimental Details

Our experiment on the RapidDiff model was completed on the NVIDIA (company headquartered in Santa Clara, California, USA) A40 computing platform with the U-Net network architecture, the Pytorch framework, and a GPU with 48 GB RAM. We selected 64 channels and 3 kernels for the CNP. During the training and testing process, our learning rate was gradually increased from 2 × 10^−6^ to 5 × 10^−5^ and remained unchanged. The number of diffusion steps per image was set to 8, and the LR image through the upsampling module was set to the pre-trained RRDB [30] module. Among them, the noise scheduling of the diffusion process was determined by the hyperparameter κ and the shifting sequence $\eta_{t}$. To elaborate, the hyperparameter κ adjusts the overall noise level during the diffusion process. Equation (4) indicates that the noise level of $x_{t}$ is proportional to $\sqrt{\eta_{t}}$, where κ is the scaling factor. Song et al. [20] show that $\kappa \sqrt{\eta_{t}}$ should be small enough to satisfy $q\left(x_{1}|x_{0},y_{0}\right)\approx q\left(x_{0}\right)$. Combining the constraints of $\eta_{1}\to 0$, we must set the minimum value of $\eta_{1}$ between ${\left(0.04/\kappa \right)}^{2}$ and 0.001. When the diffusion step reaches the final step *T*, we set $\eta_{T}$ = 0.999 to meet the requirements of $\eta_{T}\to 1$. Regarding the setting of the intermediate diffusion time steps {2,…,T−1}, we satisfy the scheduling of $\sqrt{\eta_{t}}$ as follows:

(12) $$\sqrt{\eta_{t}}=\sqrt{\eta_{1}}\times b_{0}^{\beta_{t}},t=2,\cdots ,T-1$$

where

(13) $$\beta_{t}={\left(\frac{t-1}{T-1}\right)}^{p}\times \left(T-1\right)$$

(14) $$b_{0}=\exp\left[\frac{1}{2(T-1)}\log\frac{\eta_{T}}{\eta_{1}}\right]$$

In other words, $\eta_{t}$ can be represented by $\beta_{t}$ as

(15) $$\eta_{t}=\eta_{1}\times \exp\left[\frac{\beta_{t}}{T-1}\log\frac{\eta_{T}}{\eta_{1}}\right]=\eta_{1}\times \exp\left[{\left(\frac{t-1}{T-1}\right)}^{p}\log\frac{\eta_{T}}{\eta_{1}}\right]$$

Moreover, the our selection of $\beta_{t}$ and $b_{0}$ is based on the assumption that $\beta_{1}=0$, $\beta_{T}=T-1$, and $\sqrt{\eta_{t}}=\sqrt{\eta_{1}}\times b_{0}^{\beta_{t}}$. We can infer that the rate of $\eta_{t}$ is controlled by the hyperparameter *p*. Therefore, the diffusion rate throughout the entire diffusion process can be controlled by two hyperparameters, κ and *p*.

### 4.3. Performance

In order to evaluate the images generated by image SR, we selected the PSNR, structural similarity index measure (SSIM), and LPIPS as the main quality indicators.

**ImageNet.** Table 2 shows the quantitative results of the RapidDiff model on the ImageNet dataset. We compare our RapidDiff model with eight existing models, namely ESRGAN, RealSR-JPEG, BSRGAN, SwinIR [31], Real-ESRGAN, DASR [32], LDM, and ResShift [33], in the same device experiment. The results indicate that our RapidDiff improves the PSNR and SSIM by 0.501 and 0.0295, respectively, compared to the baseline model, ResShift. Our proposed RapidDiff achieves improvements in clarity and similarity in the PSNR and SSIM but decreases the LPIPS. The PSNR is usually used to evaluate the quality of reconstructed or generated images, and a higher PSNR value means less distortion and better quality. The PSNR is increased by 0.501, indicating that RapidDiff generates images with less noise and higher overall quality compared to the benchmark model, ResShift. The SSIM is an indicator that evaluates the perceived quality of an image by comparing the brightness, contrast, and structure. The moderate increase in the SSIM value indicates that RapidDiff can better preserve the structural features of the image (such as edges and textures), making the generated images visually more accurate and aesthetically pleasing. The SSIM is improved by 0.0295, indicating that RapidDiff performs better than ResShift in preserving the structural features of images. By comparison, ESRGAN may produce artifacts in certain image SR processes, while RealSR-JPEG and SwinIR perform poorly in generating detailed and accurate textures during the image SR process. ESRGAN, BSRGAN, and Real-ESRGAN, based on GAN generation models, have acceptable performance regarding the PSNR and SSIM, possibly due to their use of PSNR-oriented loss functions, but they achieve lower LPIPS values. In addition, utilizing the advantages of the DDPM structure, LDM and ResShift outperform other existing methods in terms of the PSNR and SSIM.

**Alsat-2B.** Table 3 shows the quantitative results of the RapidDiff model on the Alast dataset. We compare our RapidDiff model with eight existing methods, namely NLSN [34], SRGAN, Beby-GAN, ESRGAN, DIT [35], EDiffSR [36], SRDiff [37], and ResShift, in the same device SR experiment. The results show that our RapidDiff improves the PSNR and SSIM by 0.162 and 0.006 compared to the baseline model, ResShift, respectively. According to the experimental results, the SR images restored by discriminative models such as NLSN are too smooth and blurry. In contrast, LR image-based generation models display higher clarity by predicting and generating information. However, this generative model frequently faces challenges such as a lack of controllability, an imbalance between diversity and authenticity in the generation process, and difficulties in cross-modal generation. Notably, in comparison to discriminative models, Beby-GAN and ESRGAN demonstrate less satisfactory perceptual quality. This shortcoming is primarily due to the excessive computational burden associated with pixel-level losses in these GAN-based frameworks. Finally, compared with the GAN and discriminative models, the two models based on the DDPM, namely ResShift and RapidDiff, generate clearer and more detailed SR images. However, our proposed RapidDiff exhibits better detail features and recovery capabilities.

### 4.4. Visual Comparisons

**Figure 7:**
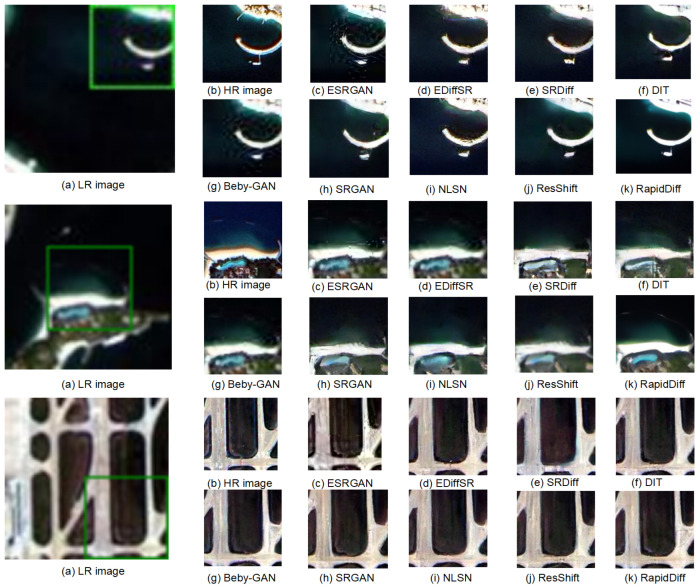
The inference results on the Alsat-2B dataset. The green box represents the image reconstruction section.

To more effectively and intuitively demonstrate the superiority of our method, we conducted a visual comparison on the aforementioned dataset. Figure 7 presents the SR reconstruction results from the Alast-2B dataset. In the comparison, the reconstruction outcomes produced by our RapidDiff approach closely align with the distribution of high-resolution (HR) images and it is capable of recreating more intricate texture details than other methods. This observation underscores the stability and applicability of our method in real-world applications.

The image reconstruction results in Figure 7 indicate that ESRGAN may produce artifacts and noise in certain image reconstructions. Due to the deep hierarchy of the ResShift network, its reconstruction quality is superior to that of ESRGAN. RealSR-JPEG, BSRGAN, SWINR, and Real-ESRGAN can produce blurry results and unwanted artifacts. The generation results of DASR, LDM, and ResShift are relatively good, but the degree of detail recovery is still not as high as that of RapidDiff.

### 4.5. Ablation Study

Table 4 and Table 5 present the results of the ablation experiment on the ImageNet dataset and Alsat-2B dataset, conducted to evaluate the performance of different conditional noise predictors composed of the U-Net single decoder, Transformer single decoder, U-Net–Transformer dual decoder, and feature fusion block on the image SR reconstruction results. The initial two rows of Table 4 and Table 5 present the outcomes of the super-resolution (SR) task conducted by the CNP utilizing a single decoder, i.e., either U-Net or Transformer. The third row illustrates the results obtained for the SR task executed by the CNP employing the dual decoder of U-Net and Transformer. Finally, the last row displays the outcomes of the SR task carried out by the CNP that integrated the U-Net–Transformer dual decoder alongside the feature fusion block. The experimental results indicate that, compared to a single decoder (U-Net or Transformer), the U-Net–Transformer dual decoder can better enhance the detailed features and texture information of image SR reconstruction, and the addition of feature integration blocks further enhances the detail reconstruction capabilities in SR tasks.

Specifically, compared to a single decoder, the CNP with dual decoders exhibits more impressive performance on the Alsat-2B dataset. The values of the PSNR and SSIM increased by 0.151 and 0.0127, and the LPIPS decreased by 0.0037. The addition of the feature fusion block further improved the performance in terms of the PSNR, SSIM, and LPIPS, increasing them by 0.197, 0.0319, and 0.0092, respectively.

### 4.6. Noise Prediction Performance

**Figure 8:**
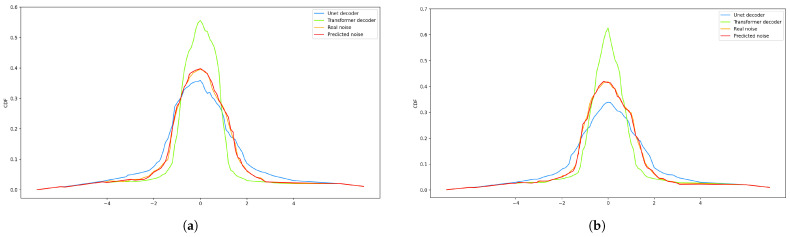
(**a**) The RapidDiff model’s noise prediction ability on the Alsat_2B dataset. (**b**) The RapidDiff model’s noise prediction ability on the ImageNet dataset.

Examples of the cumulative distribution functions (CDFs) of the Transformer decoder output and the U-Net decoder output and the real noise and anticipated noise are shown in Figure 8a,b. The CDF of the predicted noise produced by RapidDiff is nearly the same as the CDF of the real noise, despite the fact that all of the CDFs in Figure 8a,b roughly follow a Gaussian distribution. In addition, compared to the output of the Transformer decoder, the CDF output of the U-Net decoder exhibits greater variance. This is due to the fact that U-Net decoders typically provide more prominent feature information, and Transformer decoders provide more homogeneous feature information. Therefore, by combining the U-Net decoder and Transformer decoder to form a CNP, better performance can be achieved in terms of pixel-level metrics and visual perception, generating more detailed features and textures.

### 4.7. Computational Complexity Analysis

Finally, taking into account variables like the model complexity, memory usage, parameter count, and inference speed, we assessed and contrasted the computational complexity of each approach. The floating-point operations per second (FLOPs) is often used as an evaluation criterion regarding model complexity. In this study, the giga floating-point operations per second (GFLOPs), where $1GFLOPs=10^{9}FLOPs$, is used in place of FLOPs. Here, megabytes (MB), millions (M), and frames per second (FPS) represent the GPU’s memory, number of parameters, and inference speed, respectively.

The model complexity statistics for the models compared in this research are displayed in Table 6. The findings in Table 6 show that the DDPM-based image generation model has greater computational complexity than the discriminative model and the GAN-based model, which leads to a much slower inference speed. Notably, our suggested RapidDiff model outperforms previous DDPM-based models (such as EDiffSR and SRDiff) in terms of the inference time and computational complexity.

## 5. Conclusions

In this article, a new image SR reconstruction technique called RapidDiff is proposed. RapidDiff uses a CNP to optimize the noise prediction part of the denoising diffusion probability model. RapidDiff uses iterative denoising to perform image SR reconstruction and obtain the output image. The efficiency and performance of RapidDiff in image SR reconstruction were validated through experiments on the ImageNet dataset and Alsat-2B dataset. The qualitative comparison shows that RapidDiff can achieve the effects of autoregressive and generative adversarial models with fewer diffusion time steps. After the experimental analysis, when we set the diffusion step to 8, we could accelerate the inference speed while ensuring the completeness of the generated image details and clear textures. By fusing the high- and low-frequency information of the U-Net decoder and Transformer decoder through RapidDiff, the predicted noise could reach the standard of a true noise distribution, and the details and texture features of the image could be quickly reconstructed during the inference process.

Regarding the research focus of our future work, we will focus on reducing the complexity of RapidDiff’s application on large-scale datasets and increasing its computational efficiency, as well as applying it to other video and image generation tasks and ensuring convenience for downstream tasks.

## Figures and Tables

**Table 1:** Summary of ImageNet dataset and Alsat-2B dataset.

Dataset	Training Pairs	Testing Pairs	Scale Factor	Resolution
ImageNet	2800	200	4	256
Alsat-2B	2182	282	4	256

**Table 2:** Quantitative comparison on ImageNet dataset. The best and second-best results are bolded and underlined.

Method	PSNR	LPIPS	SSIM
ESRGAN	20.631	0.4872	0.4424
RealSR-JPEG	23.083	0.3227	0.6021
BSRGAN	24.372	0.2503	0.6694
SwinIR	24.021	0.2392	0.6724
Real-ESRGAN	24.121	0.2525	0.6631
DASR	24.685	0.2472	0.6821
LDM	24.932	0.2712	0.6691
ResShift	25.031	**0.1843**	0.6723
RapidDiff	**25.532**	0.2614	**0.7018**

**Table 3:** Quantitative comparison on Alsat-2B dataset. The best and second-best results are bolded and underlined.

Method	PSNR	LPIPS	SSIM
NLSN	15.660	0.4206	0.2661
SRGAN	15.675	0.3970	0.2654
Beby-GAN	**15.737**	0.3945	0.2684
ESRGAN	12.781	0.3482	0.1763
DIT	14.275	0.3621	0.2524
EDiffSR	13.519	0.1832	0.1726
SRDiff	13.852	**0.1698**	0.2115
ResShift	13.763	0.2742	0.4486
RapidDiff	13.925	0.3324	**0.4546**

**Table 4:** Ablation study on the ImageNet dataset.

U-Net Decoder	Transformer Decoder	Feature Fusion Block	PSNR	LPIPS	SSIM
✓			25.103	0.2751	0.6314
	✓		25.212	0.2874	0.6472
✓	✓		25.457	0.2632	0.6989
✓	✓	✓	25.532	0.2614	0.7018

**Table 5:** Ablation study on the Alsat-2B dataset.

U-Net Decoder	Transformer Decoder	Feature Fusion Block	PSNR	LPIPS	SSIM
✓			13.728	0.3416	0.4227
	✓		13.832	0.3461	0.4253
✓	✓		13.879	0.3453	0.4354
✓	✓	✓	13.925	0.3324	0.4546

**Table 6:** Computational complexity analysis of all methods.

Method	Complexity (GFLOPs)	Memory (MB)	Parameters (M)	Speed (FPS)
NLSN	733.69	6877	44.75	0.774
SRGAN	14.69	1653	0.73	0.802
Beby-GAN	399.71	10318	23.17	0.907
ESRGAN	9.97	1537	0.62	0.826
DIT	225.16	6356	33.13	0.005
EDiffSR	174.61	1954	30.39	0.073
SRDiff	186.08	2842	11.66	0.014
RapidDiff	100.30	452.09	118.51	0.079

## Data Availability

The ImageNet and Alsat_2B data used in this article are from https://www.image-net.org/ (accessed on 28 April 2024) and https://github.com/achrafdjerida/Alsat-2B (accessed on 15 August 2024), respectively.
